# Efficacy of Low-Level Laser Therapy in the Management of Tinnitus due to Noise-Induced Hearing Loss: A Double-Blind Randomized Clinical Trial

**DOI:** 10.1155/2013/596076

**Published:** 2013-10-28

**Authors:** Abolfazl Mollasadeghi, Seyyed Jalil Mirmohammadi, Amir Houshang Mehrparvar, Mohammad Hossein Davari, Pedram Shokouh, Mehrdad Mostaghaci, Mohammad Hossein Baradaranfar, Maryam Bahaloo

**Affiliations:** ^1^Department of Occupational Medicine, School of Medicine, Shahid Sadoughi University of Medical Sciences, P.O. Box 89138-14389, Yazd, Iran; ^2^Cardiovascular Research Center, Isfahan Cardiovascular Research Institute, Isfahan University of Medical Sciences, P.O. Box 81465-1148, Isfahan, Iran; ^3^Department of Otolaryngology and Head and Neck Surgery, School of Medicine, Yazd University of Medical Sciences, P.O. Box 89138-14389, Yazd, Iran; ^4^Industrial Diseases Research Center, School of Medicine, Shahid Sadoughi University of Medical Sciences, P.O. Box 89138-14389, Yazd, Iran; ^5^Shahid Rahnamoun Hospital, Farrokhi Ave, Yazd, Iran

## Abstract

*Background*. Several remedial modalities for the treatment of tinnitus have been proposed, but an effective standard treatment is still to be confirmed. In the present study, we aimed to evaluate the effect of low-level laser therapy on tinnitus accompanied by noise-induced hearing loss. *Methods*. This was a double-blind randomized clinical trial on subjects suffering from tinnitus accompanied by noise-induced hearing loss. The study intervention was 20 sessions of low-level laser therapy every other day, 20 minutes each session. Tinnitus was assessed by three methods (visual analog scale, tinnitus handicap inventory, and tinnitus loudness) at baseline, immediately and 3 months after the intervention. *Results*. All subjects were male workers with age range of 30–51 years. The mean tinnitus duration was 1.85 ± 0.78 years. All three measurement methods have shown improved values after laser therapy compared with the placebo both immediately and 3 months after treatment. Laser therapy revealed a U-shaped efficacy throughout the course of follow-up. Nonresponse rate of the intervention was 57% and 70% in the two assessment time points, respectively. *Conclusion*. This study found low-level laser therapy to be effective in alleviating tinnitus in patients with noise-induced hearing loss, although this effect has faded after 3 months of follow-up. This trial is registered with the Australian New Zealand clinical trials registry with identifier ACTRN12612000455864).

## 1. Introduction

Tinnitus is defined as a sound in the ear(s) without any external auditory stimulus. About 15% of the general population experience at least one episode of tinnitus, which prevalence increases by age and reaches 85% in individuals older than 60 years [1]. This symptom is intolerable in nearly 20% of the cases [2]. Reaching as high as 67%, tinnitus is more prevalent among individuals suffering from hearing disorders [3].

Noise has such deleterious effects on hearing as noise-induced hearing loss (NIHL) is the second most common form of acquired hearing loss. It has long been recognized as a problem in noisy environments workers [4]. As a possible complication of NIHL, tinnitus is usually observed at frequencies equal to or higher than 3000 Hz, which is one octave band higher than the frequencies affected in NIHL. Its intensity is usually between 3 and 5 dB (occasionally up to 15 dB) [5].

Tinnitus may lead to such complications as depression, irritability, sleep disorders, and loss of concentration [6]. Although lacking a widely accepted treatment, various therapeutic modalities have been proposed thus far, including medications (such as sedatives, antiepileptics, antidepressants, antipsychotics, local anesthetics, antihistamines, and botulinum toxin A) [7], repetitive transcranial magnetic stimulation [8], transcutaneous electrical stimulation [9], and sound therapy [10]. Low-level laser therapy (LLLT) has recently been tried with promising results in outpatients with subjective tinnitus [2].

As known, laser has different usages in medicine such as wound healing, nerve and tissue repairing, pain control [11], and treating Meniere's disease and tinnitus [12]. Although the exact mechanism of the effect of LLLT on tinnitus is not clearly understood, it has been proposed that it may be induced by increasing cell proliferation, growth factor secretion, improvement in inner ear blood flow, and/or activation of the hair cells mitochondria [2]. There is still some degree of controversy concerning the efficiency of LLLT in tinnitus. Some studies have shown positive effects [2, 11, 13, 14], but others have found no such effectiveness [15, 16].

Considering the fact that NIHL is a common disorder in industrial settings and tinnitus is its most common associated subjective complaint, we designed an interventional study to evaluate the effect of LLLT on tinnitus accompanied by NIHL.

## 2. Methods

### 2.1. Study Design and Population

The present study was a double-blind randomized clinical trial with the participation of patients referred to the occupational medicine clinic of Shahid Sadoughi University of Medical Sciences. Recruitment took place from September 2010 till September 2011.

One hundred volunteers younger than 50 years suffering from NIHL (defined as a bilateral sensorineural hearing loss, with the hearing threshold higher than 15 dB at least at one of the following frequencies: 3000, 4000, and 6000 Hz [4]) and tinnitus have enrolled to the study. The level of effect observed in a former study was used for the calculation of the sample size [2].

After baseline screening interview and examination, eleven participants were excluded from the study, yielding a final sample size of 89. Our main exclusion criteria were as follows: any history of exposure to ototoxic drugs/substances, psychotic disorders with auditory hallucination, acoustic trauma, head trauma, mumps, meningitis, Meniere's disease, and having any contraindication for laser therapy [17].

Subjects were randomly allocated to either laser therapy or placebo groups. Randomization was done using a random digit table. According to the principles of double blindness, the study participants and operators who performed the assessment tests as well as the researchers who evaluated the outcomes were completely blinded to the groups.

After taking a thorough medical and occupational history, the microscopic examination of auditory meatus and tympanic membrane was performed. Afterwards, subjects underwent pure-tone audiometry performed at 250, 500, 1000, 2000, 3000, 4000, 6000, and 8000 Hz frequencies (device: clinical audiometer, Interacoustic, AC40; headphone: TDH39, Denmark) in an acoustic chamber meeting the American National Standards Institute criterions [18]. Tympanometry was also accomplished for all participants (device: Tympanometer, Interacoustic, AZ26, Denmark). Subjects in the intervention group underwent laser therapy for 20 sessions, every other day, 20 minutes each session, which was a combination of protocols used in the previous studies [1, 2, 13]. A low-level laser beam with wave length of 650 nm and intensity of 5 mW was irradiated to the ear via mastoid bone (device: TINNImed, Switzerland). This device was connected to the ear by a soft silicone tip. The treatment sessions were performed for the subjects in placebo group with turned-off device.

A written informed consent was obtained from all participants before the enrolment. The protocol of the study was approved by the ethics committee of research vice chancellor of Shahid Sadoughi University of Medical Sciences.

### 2.2. Efficacy Assessments

We used the following three validated methods for the evaluation of outcome before treatment, immediately and 3 months after the termination of treatment: tinnitus visual analog scaling (VAS), tinnitus handicap inventory (THI), and tinnitus loudness measurement. Visual analog scale is scored on a 10-point scale, in which individuals select the lowest perceived loudness on a scale of 0 to 10 corresponding to an increasing level of loudness [19]. In THI scoring, 25 questions are asked from the patient and the severity of tinnitus is categorized as follows. Grade 1 (0–16): Slight (only heard in quiet environments); Grade 2 (18–36): Mild (easily masked by environmental sounds and easily forgotten with activities); Grade 3 (38–56): Moderate (noticed in the presence of background noise, although daily activities can still be performed); Grade 4 (58–76): Severe (almost always heard, leads to disturbed sleep patterns and can interfere with daily activities); Grade 5 (78–100): Catastrophic (Always heard, disturbed sleep patterns, difficulty with any activities) [20]. We used a translated version of the questionnaire into Persian, which was reviewed and modified by three experts to adapt our population culture. Loudness and frequency of tinnitus was assessed by audiometer. Pitch was matched by introducing two successive tones to the ear and the patient chose which one was closest to the tinnitus pitch. The loudness was assessed by matching it with the loudness of pure tone at each frequency in the contralateral ear according to the patient's sensation.

### 2.3. Statistical Analysis

Data were analyzed by the Statistical Package for Social Sciences software version 15.0 (SPSS Inc, Chicago, Illinois, USA). We used independent-sample *t*-test for the comparison of mean tinnitus loudness between two groups in three occasions (baseline, immediately, and 3 months after intervention), and paired *t*-test for the comparison of treatment effect within each group in different occasions. Chi square test was also employed in the comparison of VAS and THI score changes between two groups.

## 3. Results

**Figure 1:**
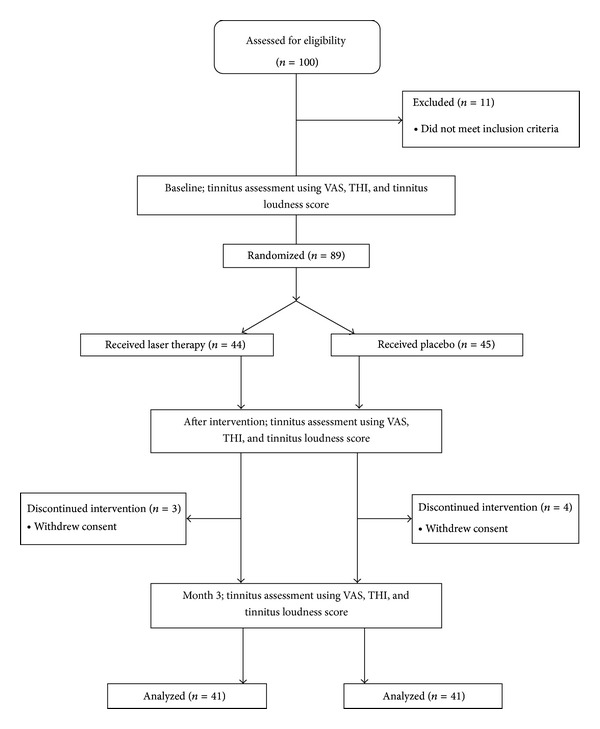
Study flow diagram.

From 100 patients screened, 89 individuals were eligible for enrolment. Reasons for exclusion were as follows: exposure to ototoxic substances (*n* = 6), head injury (*n* = 2), consumption of ototoxic drug (*n* = 1), head trauma (*n* = 1), and childhood infection (*n* = 1).  Figure 1 shows the flow diagram of the study. As demonstrated, 3 laser therapy- and 4 placebo-assigned participants have discontinued the trial due to personal reasons. Notably, no case of LLLT-attributable side effects was observed in our course of study.

All cases were males with age range of 30 to 51 years (mean: 41.17 ± 5.89 years). Their mean duration of employment was 12.21 ± 1.77 years. Mean level of noise in the workplace (time weighted average for an 8-hour shift) was 87.60 ± 1.49 dBA. Tinnitus was bilateral in 49% of the cases, while 27 and 24 percent of subjects suffered from unilateral tinnitus in left and right ears, respectively. The mean tinnitus duration was 1.85 ± 0.78 years. As expected, there was not significant difference in terms of age (*P* = 0.88), employment duration (*P* = 0.83), workplace noise level (*P* = 0.78), and duration of tinnitus (*P* = 0.62) between two randomized study groups.

Participants were categorized based on the level of experienced changes in the severity of tinnitus quantified by VAS and total THI.  Table 1 summarizes the results of between-group analyses of distribution of changes in different time intervals. As shown, LLLB was significantly more effective than placebo immediately and 3 months after treatment, which points to the efficacy of the study intervention. Nevertheless, tinnitus severity remained unchanged in 54% and 70% of patients immediately and 3 months after receiving LLLB (as measured by VAS).

According to Table 2, tinnitus loudness scores were comparable between two groups at baseline. After receiving LLLB, tinnitus loudness score was diminished in a U-shape manner with significantly lower scores than placebo in all time points.

Changes in tinnitus loudness score were compared within and between groups in different time periods. As expressed in Table 3, LLLB reduced the loudness of tinnitus significantly in relation to the baseline values and compared with the placebo group in all time periods. A meaningful response was also detected in placebo-assigned individuals immediately after treatment, which still was significantly lower than that of the intervention group.

## 4. Discussion

Previously published studies have reported the efficacy of LLLT in decreasing tinnitus to be between 15–67% [11]. Quantifying by VAS, our positive findings have been multiplied by some [2, 11, 13], while negated by other studies [15, 21]. Tauber et al. used 10 sessions of LLLT with two different wavelengths (635 and 839 nm) during two weeks which was different from our practice [11]. Okhovat et al. were treated patients with 20-minute sessions a day for 20 days using the same wavelengths to our study [2]. The most similar protocol to ours was used by Yıldırım et al., with considerable improvements which sustained after two months [13].

Mixed results were also obtained by studies that have used tinnitus loudness score as their primary outcome measure. While this study in line with Tauber et al. [11], Gungor et al. [22], Newman et al. [20], and Shiomi et al. [23] has found tinnitus loudness to be improved after LLLT, two evaluations have failed to show the same efficacy [15, 16]. A noteworthy point to consider is the pronounced improvement reported by our patients after receiving placebo, which vanished at the end of the follow-up period. This observation, to our opinion, might be explained best by a placebo effect.

The results of total THI score in our study were in accordance with VAS results after LLLT and were consistent with the study of Cuda and Caria [14], but Teggi et al. did not show this change [15].  Table 4 presents a detailed comparison between the findings of some relevant studies with what we found in our population. We suppose that the controversial results could be attributable to employing different treatment courses, as well as varied experiment settings. For instance, our patients received therapy in clinic, while Teggi et al. [15] gave the participants their course of treatment at home.

Even though it remained higher compared with the baseline level and placebo group, we observed that the effect of LLLT attenuated after 3 months. Our finding was attenuated by another research with assessment period of 4 weeks and 6 months [11]. It seems that the efficacy of LLLT decreases over time, which may necessitates repeating the therapy. Further evidence, however, is needed for determining a proper time interval between sessions.

While most of the former comparable studies have not taken concomitant hearing disorders into consideration, we assessed the effect of LLLT on tinnitus in a background of sensorineural hearing loss. However, our results should be interpreted in the light of some limitations. The first limitation was our 3-month follow-up period that made it impossible to evaluate long-term outcomes of the studied intervention. Secondly, due to the fact that our study population comprised of male workers, the obtained results may hardly be generalized to other populations.

In conclusion, this study has provided evidence for the efficacy of LLLT in relieving NIHL accompanied tinnitus, an effect that was weakened after 3 months follow-up. Despite significant improving results, the LLLB treatment nonresponse rate was considerable which should be taken into account when considering this treatment method.

## Figures and Tables

**Table 1:** Comparison of changes in tinnitus visual analog scaling and tinnitus handicap inventory scores immediately and 3 months after intervention between groups.

Variable [number (%)]	Immediately after intervention			3 months after intervention		
	Laser therapy group	Placebo group	*P* value	Laser therapy group	Placebo group	*P* value
Visual analog scale score						
No difference	22 (54)	35 (85)	0.006	29 (70)	40 (97)	0.003
<50% reduction	7 (17)	3 (7.5)		5 (13)	1 (3)	
≥50% reduction	12 (29)	3 (7.5)		7 (17)	0 (0)	

Tinnitus handicap inventory score						
No difference	21 (51)	36 (87)	0.001	27 (66)	40 (97)	0.001
<50% reduction	2 (6)	1 (3)		1 (3)	0 (0)	
≥50% reduction	18 (43)	4 (10)		13 (31)	1 (3)	

**Table 2:** Comparison of tinnitus loudness between two groups at baseline, immediately and 3 months after intervention.

Tinnitus loudness (dB)	Placebo	Laser therapy group	*P* value
Baseline	6.09 ± 1.11	6.07 ± 1.12	0.922
Immediately after intervention	5.97 ± 1.03	4.51 ± 1.89	<0.001
3 months after intervention	6.02 ± 1.15	5.09 ± 1.90	0.009

*Data are presented as mean ± standard deviation.

**Table 3:** Comparison of the changes of tinnitus loudness in 3 periods of assessment within and between groups.

Tinnitus loudness (dB)	Placebo		Laser		*P* value for between-group comparison
	Mean ± SD	*P* value	Mean ± SD	*P* value	
Baseline	6.09 ± 1.11	0.023	6.07 ± 1.11	<0.001	<0.001
Immediately after intervention	5.97 ± 1.03		4.51 ± 1.03		

Baseline	6.09 ± 1.11	0.183	6.07 ± 1.11	<0.001	<0.001
3 months after intervention	6.02 ± 1.15		5.09 ± 1.15		

Immediately after intervention	5.97 ± 1.03	0.421	4.51 ± 1.03	0.013	0.01
3 months after intervention	6.02 ± 1.15		5.09 ± 1.15		

**Table 4:** Comparison of the design and results of some relevant studies with the present study.

Author (Year)	Number of cases	Laser properties			Placebo controlled	Followup	Tinnitus scoring method			Effect of laser in treatment
		Wave length	Power	Duration of treatment			VAS	THI	Loudness	
Present study	82	650	5	20 min, 3 days a week, for 20 sessions	+	+	+	+	+	Pos.
Shiomi et al. (1997) [23]	38	830	40	9 min a week for 10 sessions	−	−	−	−	+	Pos.
Mirz et al. (1999) [21]	59	830	50	10 min per session	+	−	+	+	+	Neg.
Nakashima et al. (2002) [16]	45		60	Once a week for 4 weeks	+	−	−	−	+	Neg.
Prochazka (2002) [1]	72	830	300	Twice a week for 5 weeks and after 2-3 m. 5-6 sessions in a week	−	−	−	+	−	Pos.
Tauber et al. (2003) [11]	35	G1: 635 G2: 830	G1: 7.8 G2: 20	5 days a week for 2 weeks	−	+	+	−	+	Pos.
Gungor et al. (2008) [22]	45	650	5	15 min a day for 1 week	+	+	−	−	+	Pos.
Cuda and De Caria (2008) [14]	46	650	5	20 min a day for 3 months	+	−	−	+	−	Pos.
Teggi et al. (2009) [15]	60	650	5	20 min a day for 3 months	+	+	+	+	+	Neg.
Okhovat et al. (2011) [2]	61	650	5	20 min a day for 20 days	−	−	+	−	−	Pos.
Yıldırım et al. (2011) [13]	30	650	5	20 min 5 times a week for 8 weeks	−	+	+	−	−	Pos.
